# Porphyrins Through the Looking Glass: Spectroscopic and Mechanistic Insights in Supramolecular Chirogenesis of New Self-Assembled Porphyrin Derivatives

**DOI:** 10.3389/fchem.2020.587842

**Published:** 2020-10-15

**Authors:** Manuela Stefanelli, Marco Savioli, Francesca Zurlo, Gabriele Magna, Sandra Belviso, Giulia Marsico, Stefano Superchi, Mariano Venanzi, Corrado Di Natale, Roberto Paolesse, Donato Monti

**Affiliations:** ^1^Department of Science and Chemical Technologies, University of Rome “Tor Vergata”, Rome, Italy; ^2^Department of Sciences, University of Basilicata, Potenza, Italy; ^3^Department of Electronic Engineering, University of Rome Tor Vergata, Rome, Italy; ^4^Department of Chemistry, University La Sapienza, Rome, Italy

**Keywords:** porphyrins, circular dichroism, supramolecular chemistry, supramolecular chirogenesis, self-assembly, chirality

## Abstract

The solvent driven aggregation of porphyrin derivatives, covalently linked to a L- or D-prolinate enantiomer, results in the stereospecific formation of species featuring remarkable supramolecular chirality, as a consequence of reading and amplification of the stereochemical information stored in the proline-appended group. Spectroscopic, kinetic, and topographic SEM studies gave important information on the aggregation processes, and on the structures of the final chiral architectures. The results obtained may be the seeds for the construction of stereoselective sensors aiming at the detection, for example, of novel emergent pollutants from agrochemical, food, and pharmaceutical industry.

## Introduction

**Scheme 1:**
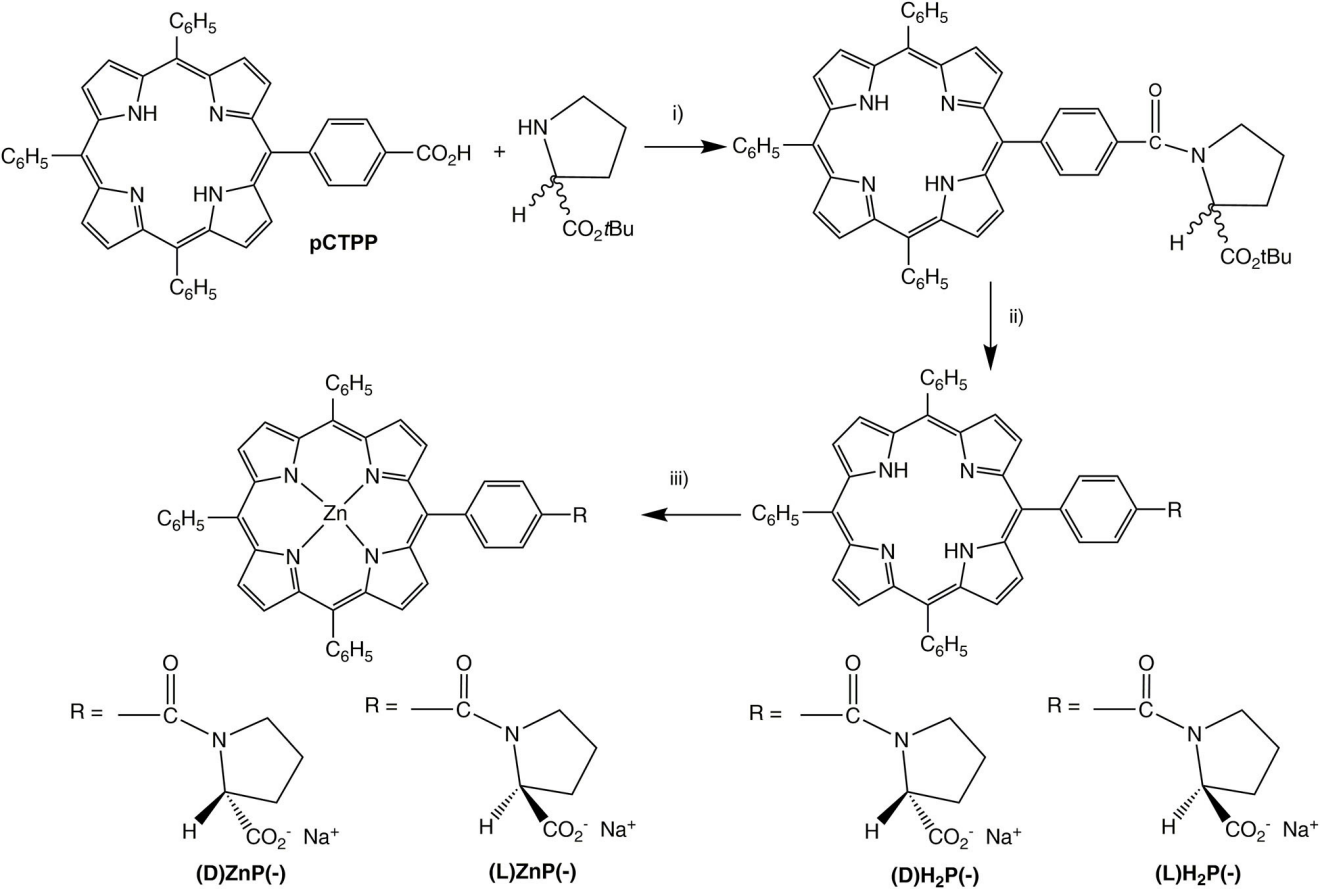
(i) EDCl, HOBT, dry CH_2_Cl_2_, 0°C, 1 h; then RT, 48 h. (ii) TFA/CH_2_Cl_2_ (2/3, v/v), 1.5 h, then aqueous NaHCO_3_. (iii) Zn(OAc)_2_·2H_2_O, CHCl_3_/MeOH, RT, 1 h.

Porphyrin-based chiral supramolecular systems are of great importance owing to their potential application in wide fields of science and technology (Cui et al., 2015; Paolesse et al., 2017; Lee et al., 2018), and for the implication on the emergence of homochirality in Life (Guijarro and Yus, 2009). Addressed protocols pursued for the formation of such a species usually rely on the use of either chiral or achiral platforms (Monti, 2014; Liu et al., 2015; Magna et al., 2019). In the former case, the stereochemical course of the self-assembling process is governed by the presence of chiral groups linked on the porphyrin peripheral frame (Oliveira-González et al., 2015; van der Weegen et al., 2017). In the latter issue, final chiral suprastructures are obtained by interaction of achiral substrates with external physical effectors, such as hydrodynamic directional forces (Sorrenti et al., 2012; Arteaga et al., 2016); magnetic fields (Micali et al., 2012); LB and LS techniques (Chen et al., 2011), or chiral molecular templates, such as surfactants (El-Hachemi et al., 2008), chiral polymeric matrices (D'Urso et al., 2012), chiral ligands (Borovkov and Inoue, 2006; Berova et al., 2007) or carboxylic acids (Castriciano et al., 2011). With this regard, Monsù Scolaro showed that the assembly of a non-chiral porphyrin derivative templated by the two different enantiomers of tartaric acid proceeds with different kinetics and results in final chiral suprastructures with highly different anisotropy factors, which are strictly dependent on the enantiomer used as a templating agent (Castriciano et al., 2012). These findings indicate the complexity of factors that play a crucial role in the formation of such chiral species. As a part of our studies dedicated to the construction of chiral porphyrin aggregates (Monti et al., 2010; Zelenka et al., 2011), we investigated the self-assembly behavior of intrinsically chiral zinc-tetraphenylporphyrin derivatives, characterized by the presence of covalently linked enantiomers of L- or D-prolinate groups on the molecular frame (Scheme 1). The molecular information stored on the periphery of the macrocycles, besides inferring key amphiphilic properties to the monomers, can be effectively read out during the self-assembly of the aromatic platforms, once steered by hydrophobic effect, owing to the conformational rigidity, and robustness toward adventitiously caused racemization of the prolinate residues. This, in certain aspects, unique aminoacid has been recently found to play a role in asymmetric aminocatalysis (Mathew et al., 2004). We may anticipate that the process follows a highly stereospecific multistep mechanism, to give assemblies with specular CD features, indicating the formation of aggregate structures with opposite supramolecular chirality, dictated by the type of stereocenter present on the macrocycle. Further kinetic and topographic SEM investigations gave clear cut insights on the mechanism and the morphology of the chiral supramolecular structures formed. The results obtained are of great importance for the fabrication of sensors featuring stereoselective properties. We recently found, for example, that hybrid organic-inorganic materials, constituted by ZnO nanoparticles covalently linked to a porphyrin enantiomer studied in detail in the present work, are able to selectively detect the different enantiomers of chiral analytes, such as limonene and other terpenes (Stefanelli et al., 2019).

## Results and Discussion

### Synthesis of Porphyrin Derivatives

Synthesis of the title porphyrins has been accomplished by following the path reported in Scheme 1, which relies on straightforward procedures used in peptide chemistry. As a first stage, the 5-(4-carboxyphenyl)-10,15,20-triphenylporphyrin **pCTPP** was reacted with (L)- or (D)-proline *tert*-butyl ester using the EDCl/HOBT coupling reagents, providing the corresponding prolinated derivatives which were subsequently hydrolyzed in a TFA/CH_2_Cl_2_ mixture to give the amphiphilic compounds **(L)H**_**2**_**P**(–) and **(D)H**_**2**_**P(–)**. The further metalation with an excess of zinc acetate in CHCl_3_/MeOH afforded the corresponding **(L)ZnP**(–) and **(D)ZnP**(–**)** complexes (Stefanelli et al., 2019).

### Aggregation Studies

#### Spectroscopic Investigations

Aggregation of title porphyrins has been carried out in aqueous ethanol solutions (EtOH/H_2_O 25:75 v:v; 298 K) typically at 5 μM concentration, by strictly following a “porphyrin first” protocol, as reported in the Experimental Section. Previously published studies, carried out by our group and by others, pointed out the strong dependence of the aggregation process on the experimental conditions, such as the order in which the reagents are mixed (Micali et al., 2000). Moreover, the chiral induction exerted by homochiral dopants can be effectively modulated during the early milliseconds mixing stage (i.e., standard flask preparation *vs*. microfluidic mixing; Sorrenti et al., 2016). However, within the standard playground of solutions preparation, the peculiar solvent mixture used allows for a good reproducibility of the results and a convenient time of reaction, to be followed by conventional spectroscopic techniques. It is important to remark that the preparation of the samples requires also an additional step of sonication and microfiltration of the solutions, to ensure an optimal reproducibility of the results obtained, in terms of spectral features (UV-Vis and CD band intensities) and kinetic parameters (for a brief discussion see Supplementary Material). Sonication steps have been demonstrated to feature a key role in supramolecular chirogenesis of porphyrin macrocycles and related structures (Liu et al., 2015; El-Hachemi et al., 2016). The protocol followed is described in full details in the Experimental Section.

**Figure 1:**
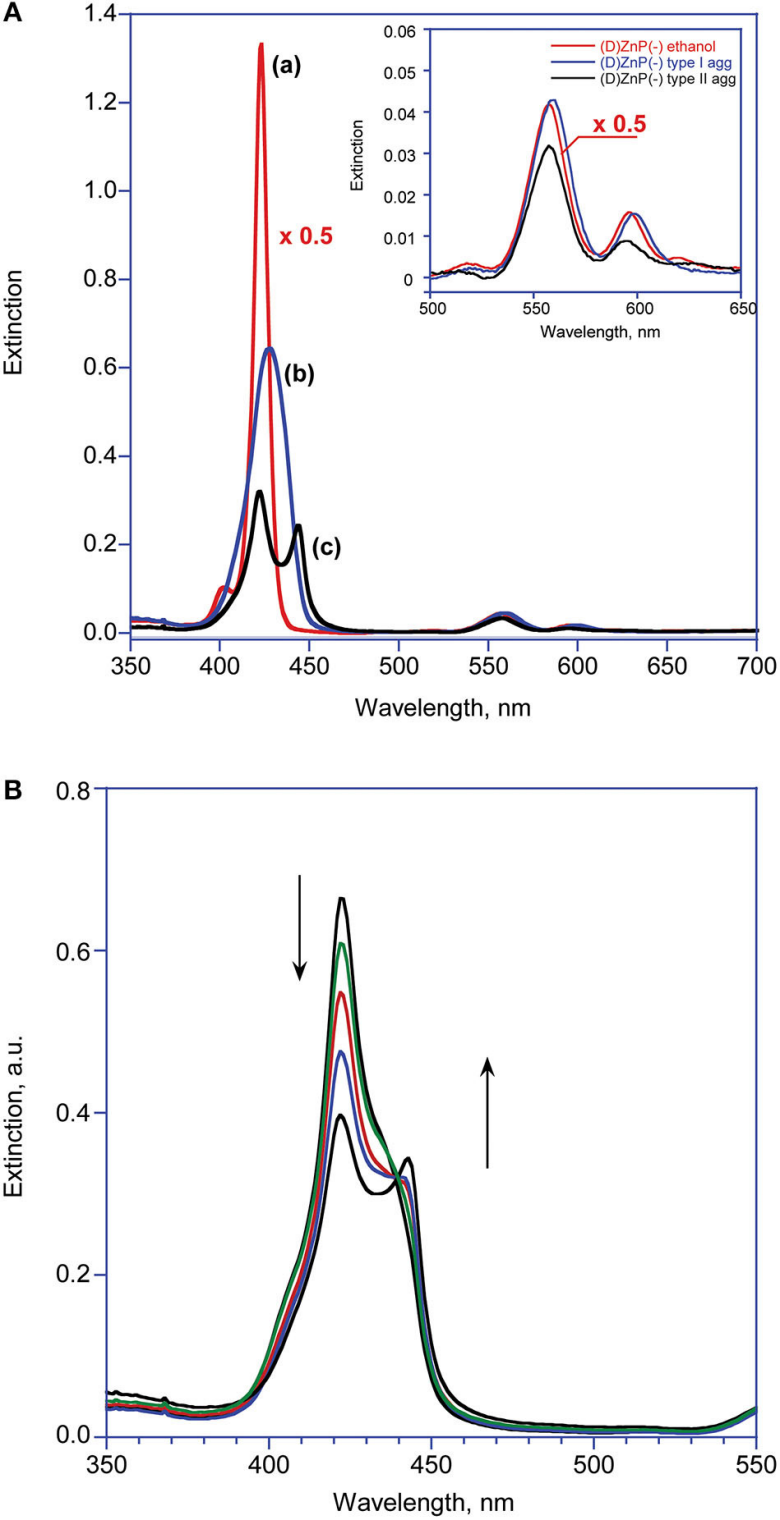
**(A)** UV-Visible spectroscopic pattern of **(D)ZnP(–)** 5.0 μM in various media at 298 K: (a) ethanol; (b) EtOH/H_2_O (25:75 v:v) after mixing (Type-I aggregates); (c) EtOH/H_2_O (25:75 v:v) at equilibrium (Type-II aggregates). The inset reports the variations of the corresponding Q bands. **(B)** Extinction vs. time evolution of Type-I into Type-II aggregates. The term “Extinction” is consistently used instead of the more usual “Absorbance,” as the intensities of the UV-Vis features are unavoidably affected by the contribution of the RLS component (Micali et al., 2001).

**Figure 2:**
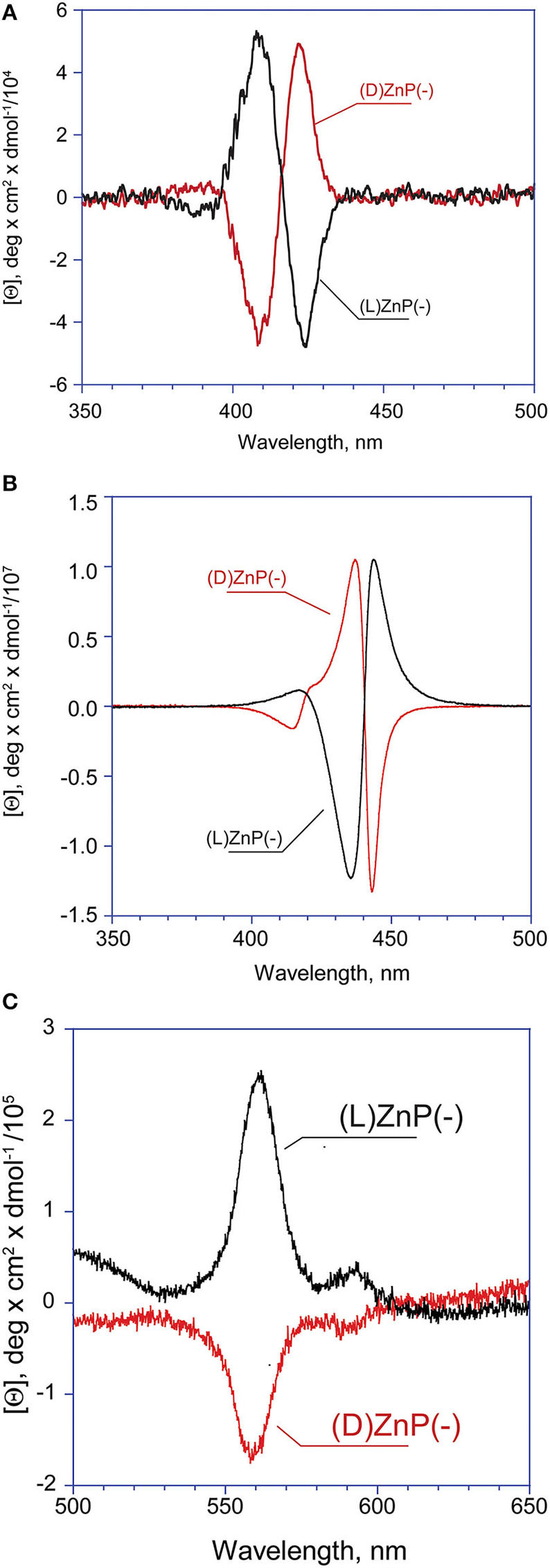
CD spectra of **(D)-** and **(L)ZnP(–)** 5 μM (EtOH/H_2_O 25:75 v:v): **(A)** Type-I aggregates (*t* = 0); **(B)** Type-II aggregates (at equilibrium). **(C)** Enlargement of the Type-II aggregate Q bands region.

In ethanol, the title amphiphilic macrocycles are in monomeric form (λ_max_ = 424 nm; Figure 1A, trace a); the addition of a proper amount of water steers the self-aggregation process driven by hydrophobic effect, as indicated by the typical UV-Vis spectral changes (Figure 1A, trace b), which is complete within the time of mixing, likely driven by π-π interactions (Type-I aggregates). The corresponding spectral pattern indicates a hypochromic effect with a concomitant broadening and small red-shift of the Soret B band to 427 nm. CD spectroscopy reveals that these species feature, although of low intensities, appreciable supramolecular chirality of opposite sign, depending on the configuration of the appended groups. In particular, the aggregates of **(D)ZnP(-)** show a positive (+/–) bisignate spectrum, whereas those of the **(L)ZnP(-)** enantiomer show a mirrored, negative pattern (–/+), as reported in Figure 2A. The reproducibility of the results, along with the specularity of the dichroic bands, safely rules out any effect due to the presence of adventitious chiral residues in the medium (El-Hachemi et al., 2009), also upon using of distilled water from different sources and time of storage. Interestingly, these species show a further slow evolution, which is generally completed within few weeks, into structures of different morphology (Type-II aggregates; Figure 1A, trace c). Accordingly, the UV-Vis spectra of the solutions show a further slow decrease of intensity of the Soret bands, evolving at final equilibrium into a double peaked coupled B band (λ_max_ = 422 and 443 nm) in the case of both **(L)ZnP(-)** and its **(D)ZnP**(**–**) stereoisomer, with a clear isosbestic point at 439 nm (Figure 1B).

Again, CD experiments gave insights on this phenomenon. In the cases of both of the enantiomers, the spectra show remarkable evolution of the initial features, with the gradual formation of multi-patterned coupled bands featuring intensities increased by two order of magnitude, with crossover points in correspondence of the UV-Vis absorption maxima (Figurse 2B,C). This complex pattern has been ascribed to the formation of J-type aggregates of complex morphology, with excitonic coupling along two preferential space directions (Ohno et al., 1993). In particular, the supramolecular structures derived from the **(L)ZnP**(–**)** species, show a +/–/–/+ pattern, while those of the **(D)ZnP(**–**)** forms show a specular –/+/+/– sign. Accordingly, the calculated anisotropy factors *g* (Berova et al., 2000) are 9.5 (± 0.5) × 10^−5^ for both the initial (Type-I) aggregates; 0.068 (± 0.005) and 0.071 (± 0.008) for **(L)ZnP(**–**)** and **(D)ZnP(**–**)** respectively, at equilibrium (Type-II species; 5 μM initial concentration). These values indicate an enhancement of the intrinsic chirality on going to the larger, more structured Type-II species.

Accordingly, Type-II aggregates showed quenching of fluorescence emission (Supplementary Figure 1A), and intense Resonance Light Scattering features (Supplementary Figures 1B,C), as a consequence of an efficient delocalization of porphyrin exciton momenta over a large number of electronically coupled monomers, with respect to those featured in the first aggregation stage, indicating the formation of larger and strongly coupled self-assembled structures (Pasternack et al., 1993). Finally, the corresponding excitation spectra, carried out at both the emission wavelength maxima (i.e., at 604 or 650 nm) gave the exact reproduction of the UV-Vis Type-II spectral pattern, ruling out the formation of distinct families of H and J aggregates (Supplementary Figure 1D). The supramolecular structures formed at 10 μM concentration featured a somewhat reduced *g*-value to about 0.049 (± 0.006), indicating a lesser degree of order of the final structures. Analogous and even more dramatic effect, has been previously reported (Romeo et al., 2014), pointing out the importance of the initial nucleation stage preceding the ordered growth of final structures.

The results obtained showed good reproducibility well within a factor of two, also with porphyrins from different synthetic batches, although a strong decrease of the overall CD spectral intensities of the aggregates is generally observed when aged porphyrins stock solutions are used (time of storage longer than 1 month). Conversely, studies carried out at 1 μM concentration did not give evidences of complete formation of Type-II chiral aggregates, resulting in corresponding dichroic bands of very low intensity, which in some cases were difficult to be disentangled from the background noise. Prolonged standing of these solutions merely resulted in the flocculation of uncharacterized material.

**Scheme 2:**
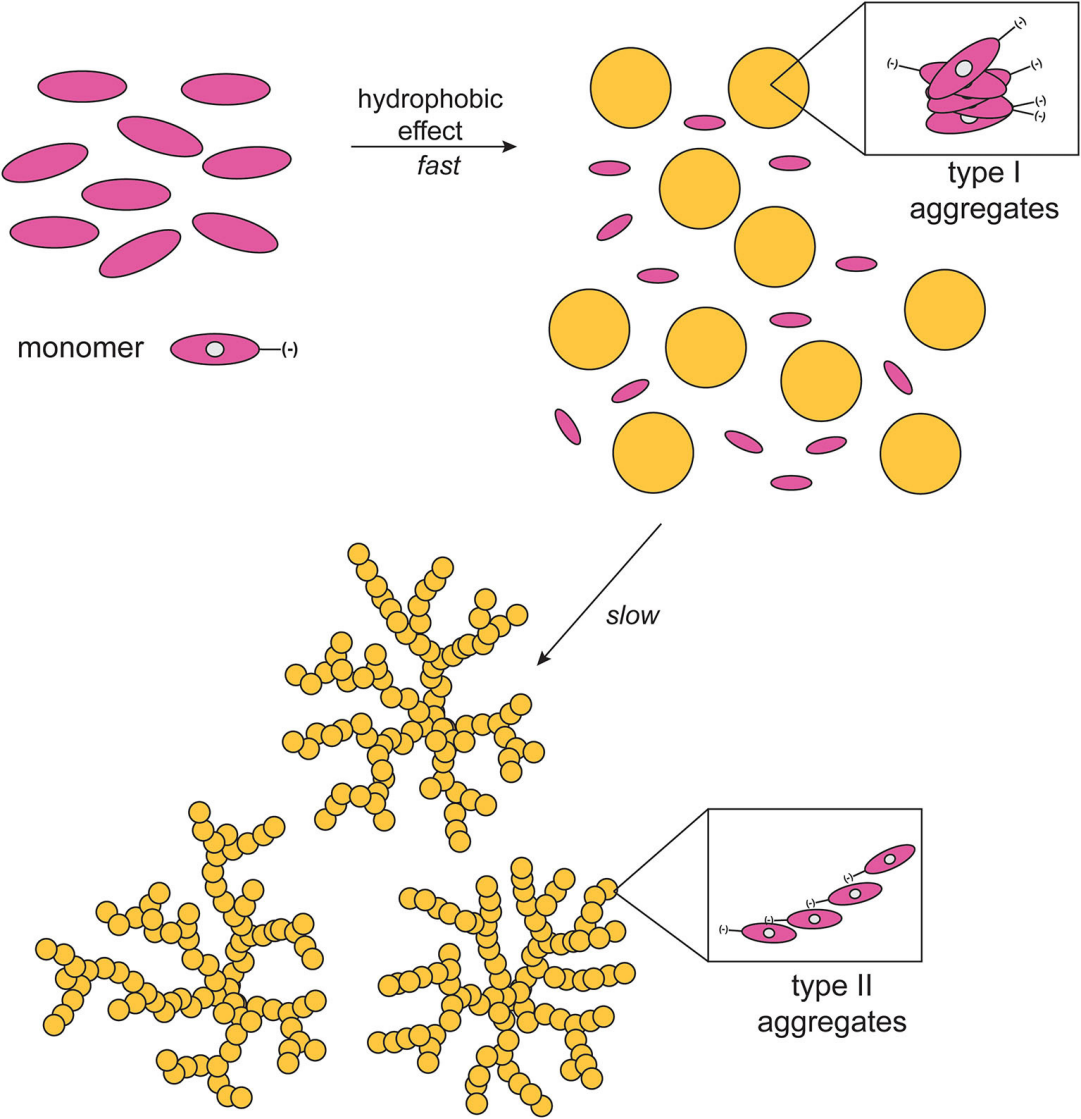
Schematic drawing of the proposed mechanism of aggregate formation (EtOH/H_2_O 25:75 v:v; T = 298 K).

It should be emphasized that in all of the cases observed the final CD spectra feature a sign inversion of the main lower energy bands, with respect to the blue-shifted coupled pattern. Inversion of these J-band has been also found by others in the case of the formation of chiral porphyrin aggregates upon symmetry breaking induced by rotary evaporation (Sorrenti et al., 2012), and has been ascribed to a distortion from the planarity of the macrocycles due to steric hindrance. Similar results have been reported by Micali and Monsù Scolaro (Micali et al., 2006), for the case of chiral fractal structures of a water-soluble tetrakis(*p*-sulphonatophenyl)porphyrin derivative, assembled in the presence of D- and L-tartaric acid as chiral effectors. In the present case, this effect may arise from a coordination of the Zn ion by the proline carboxylate group, and this event would likely constitute the driving force for the evolution toward the final Type-II species. The involvement of Zn-coordination is also suggested by the fact that the free-base analogs of the title porphyrins [**(D)**- and **(L)H**_**2**_**P(–)**; Scheme 1] show different aggregation behavior with different CD spectral features, which are composed by simple mirrored bisignated bands, with constant intensity and pattern throughout the whole temporal window examined and, relevantly, with same signs of that of the Type-I species (Supplementary Figure 2). As far as the macrocycles intimate molecular level structure is concerned, a metastable mutual *syn* configuration for the Type-I aggregates, and a thermodynamically stable extended *anti* configuration could be proposed. This suggestion was made on the basis of strong similarities of both Soret band and CD profiles of the aggregates to those of chiral *syn-* or *anti*-geometry of bis-(Zn)porphyrin derivatives with several chiral ligands (Borovkov et al., 2000, 2001). A pictorial representation of the mechanism is reported in Scheme 2. Furthermore, if the aggregation of the title Zn porphyrins is carried out in the presence of an excess of benzylamine, a competing achiral nitrogen ligand, the aggregation occurs with the formation of final achiral, CD silent, aggregate structures (results not shown), confirming the key role of the coordination on the formation of the final structures.

As far as the overall aggregation process is concerned, we recently found an analogous two-step process in the aggregation of related cationic L-proline-porphyrin derivatives in analogous aqueous media (Lorecchio et al., 2014; Caroleo et al., 2019), and also in the study of the interaction of a chiral Zn-porphyrin derivative with chiral anionic surfactants in water, that results in the rapid formation of small sized aggregates, followed by a slower step in which the initial species rearrange in more ordered and specific structures driven by Zn-coordination (Simoncini et al., 2014). Same phenomenon has been also observed by others in organic solvent (Feldborg et al., 2011), in the case of water-soluble tetrapyrrolic macrocycles (El-Hachemi et al., 2016) or, amazingly, even in solid state (Borovkov et al., 2003).

Finally, when the aggregation experiment was carried out on the racemic mixture (i.e., 5 μM of both the enantiomers) only a broadening and hypochromic effect of the Soret bands and silent CD spectra are obtained, indicating the formation of non-specific achiral structures, likely due to the mismatching of the chiral proline groups during the key molecular recognition event.

#### Kinetic Studies

**Figure 3:**
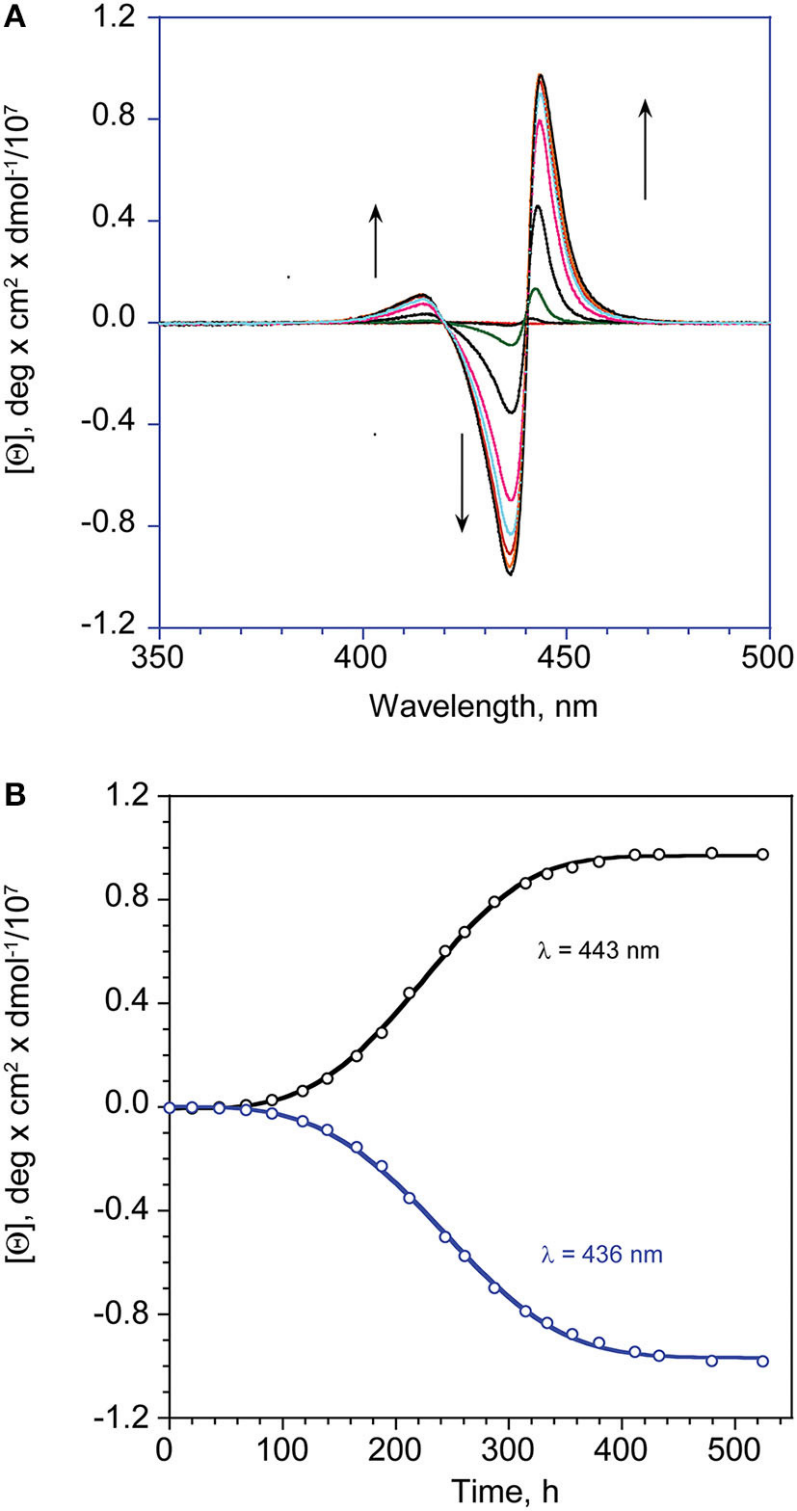
**(A)** CD spectral changes with time of **(L)ZnP(–)**, 5 μM (EtOH/H_2_O 25:75 v:v). **(B)** Corresponding calculated fit (Equation 1) at the indicated wavelength maxima of the lower energy bands.

Kinetic studies, carried out on the slow formation step of Type-II aggregates, allowed to get more insights on the intimate mechanism of formation of such important chiral architectures. In all of the case examined, the kinetic profiles [i.e., molar ellipticity *[*Θ*] vs*. time] followed a sigmoidal autocatalytic behavior (Figures 3A,B) that could be successfully modeled by a “*fractal-type*” exponential equation (Equation 1) formerly developed by Pasternack et al. (2002) for the case of disassembly of porphyrin aggregates by cyclodextrins. The equation used is of the form:

(1) $$\left[\Theta \right]={\left[\Theta \right]}_{eq}+({\left[\Theta \right]}_{o}-{\left[\Theta \right]}_{eq})e^{\left[\frac{-{\left(kt\right)}^{n+1}}{\left(n+1\right)}\right]}$$

In this equation, *[*Θ*], [*Θ*]*_0_, and *[*Θ*]*_*eq*_ are the molar ellipticity values at time t, time = 0, and at equilibrium, respectively; *n* is the “aggregate growth rate” parameter, and *k* the kinetic constant. We consistently found values of *n* > 1, indicating that the interaction between monomers and the growing structures become increasingly favored with time, i.e., with the increase of their surface area.

It is worthwhile of noting that this equation is a reduced form of a more general non-conventional equation previously developed by Pasternack himself (Pasternack et al., 2000), employed in the kinetic studies of fractal growth of porphyrin aggregates onto DNAs. This latter equation includes, beside a kinetic constant for an uncatalyzed path (*k*_0_), a further parameter (*m*) related to the number of monomers forming the aggregation nuclei with catalytic activity (nucleation seeds). The equation reduces to Equation 1 in the case of *m* = 1, implying that the rate-determining step is the adhesion of the monomer to the growing assemblies, and not the formation of catalytically-active nucleation seeds. In our case the initial oligo-assemblies formed (Type-I species) should act as *single-site enantiomorphic surfaces* (i.e., a collection of uniform and independent chiral interaction sites), that promote the catalyzed growth of the final suprastructures with high stereospecificity. The legitimacy of this hypothesis is corroborated by the fact that the fit of the experimental values with the original “*full-form equation*” always gave *m*-values very close to 1 (i.e., from 0.95 to 1.1 ± 0.2) and negligible values of *k*_0_ that faded within their experimental errors, along with a lower adherence to the experimental data points and higher uncertainties of the obtained kinetic parameters. The results have been reported in Table 1.

**Table 1:** Kinetic parameters for the aggregation reaction of **(L)ZnP(–)** and **(D)ZnP(–)** in EtOH/H_2_O (25:75 v:v; 298 K) at different concentrations.

	**(L)ZnP(–)**		***(D)ZnP(–)***	
**Concentration, M**	***k*, min^**−1**^**	***n***	***k*, min^**−1**^**	***n***
(1) 5.0 × 10^−6^ M^(a)^	9.3 (± 0.8) × 10^−5^	2.3 ± 0.7	9.0 (± 0.8) × 10^−5^	2.7 ± 0.6
(2) 5.0 × 10^−6^ M^(b)^	9.7 (± 0.6) × 10^−5^	1.8 ± 0.3	8.5 (± 0.4) × 10^−5^	1.7 ± 0.2
(3) 1.0 × 10^−5^ M^(a)^	8.4 (± 0.7) × 10^−4^	4.8 ± 0.9	7.3 (± 0.6) × 10^−4^	4.0 ± 0.6
(4) 1.0 × 10^−6^ M^(c)^	6.0 (± 0.5) × 10^−2^	0.12 ± 0.05	5.0 (± 0.4) × 10^−2^	0.11 ± 0.03

*(a) Followed by CD spectroscopy, fitted by Equation 1; (b) Followed by UV-Vis spectroscopy (λ = 423 nm), fitted by Equation 1; (c) Followed by UV-Vis spectroscopy (λ = 422 nm), fitted by Equation 2*.

From the inspection of the table, the second step kinetic constant has been found to be in the order of 10^−4^ min^−1^ for the process carried out at 5 μM, for both of the enantiomers, with *n*-values of *ca* 2 (entry 1). Kinetic experiments carried out at 10 μM concentration resulted in an increase of the rate constant within one order of magnitude, along with an exponential growing factor of *n* = 4 (entry 3), which should arise from both the increase of the number of the initial nucleation structures, and on their increased size (i.e., area of the catalytic surfaces). Same results, in good agreement within experimental errors, have been obtained by UV-Vis spectroscopy, by following the slow decay with time of the Soret band of the Type-I species (see Table 1, entry 2, and Supplementary Figure 3).

**Figure 4:**
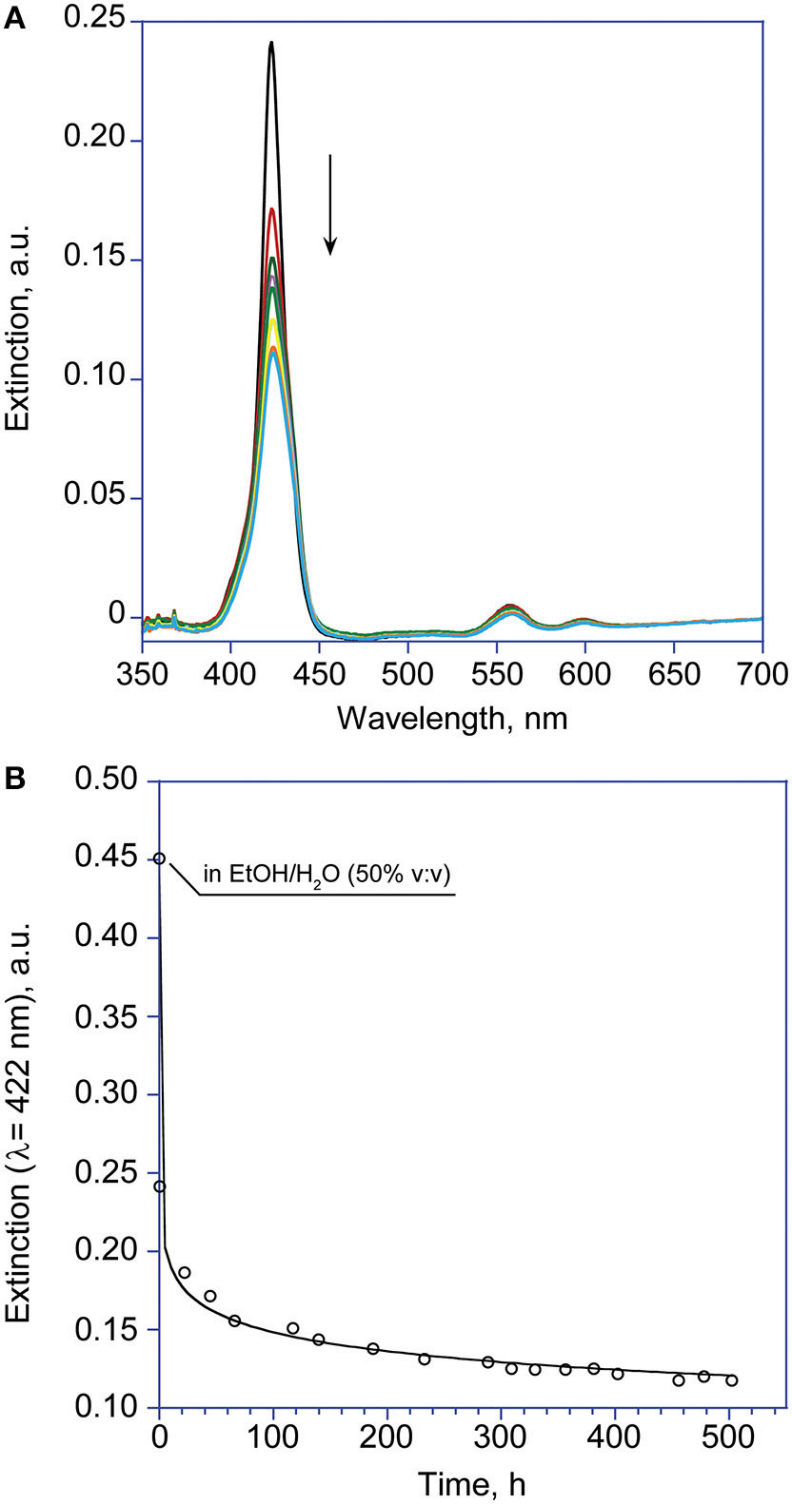
**(A)** UV-Visible spectroscopic changes with time of the Soret band of **(L)ZnP(–)** 1 μM (EtOH/H_2_O 25:75 v:v). **(B)** Corresponding calculated fit according to Equation 2 (λ = 422 nm). The point at *t* = 0 is measured in non-aggregative conditions, due to the fast rate of the process.

Interestingly, experiments carried out at 1 μM concentration revealed that the formed aggregates featured very low supramolecular chirality. However, the lower aggregation rate observed at this concentration regime, allowed for shedding light on the initial fast aggregation stage (i.e., formation of Type-I molecular structures) by UV-Vis technique. Remarkably, the *extinction vs. time* plot revealed a Diffusion Limited Cluster-Cluster Aggregation kinetic (DLCCA; Figure 4), which entails the rapid formation of small clusters, that subsequently slowly stick together forming larger structures (Monsù Scolaro et al., 2002). The equation used is of the form

(2) $$\begin{array}{r} \left[E\right]={\left[E\right]}_{o}+\left({\left[E\right]}_{eq}-{\left[E\right]}_{o}\right)\left(1-e^{{\left(-kt\right)}^{n}}\right) \end{array}$$

where *E* is the extinction values of the Soret band of the monomeric forms (λ = 424 nm) at time t, at time = 0 and at equilibrium, respectively; *k* is the apparent first-order reaction constant, and *n*, the so called “stretching factor,” modulates the diffusion growth of the aggregates, which in the case of this observed mechanism is strictly required to be *n* < 1. Due to the fast decay of the Soret intensity, the value at time *t* = 0 is calculated in the closest non-aggregative conditions (EtOH/H_2_O 50% v:v).

The corresponding fit gave an estimation of the calculated rate constant values in the order of 10^−2^ min^−1^, whereas the *n*-values are about 0.1 for both **(L)ZnP**(**–**) and **(D)ZnP**(**–**) (entry 4, Table 1).

### Topographic SEM Investigations

**Figure 5:**
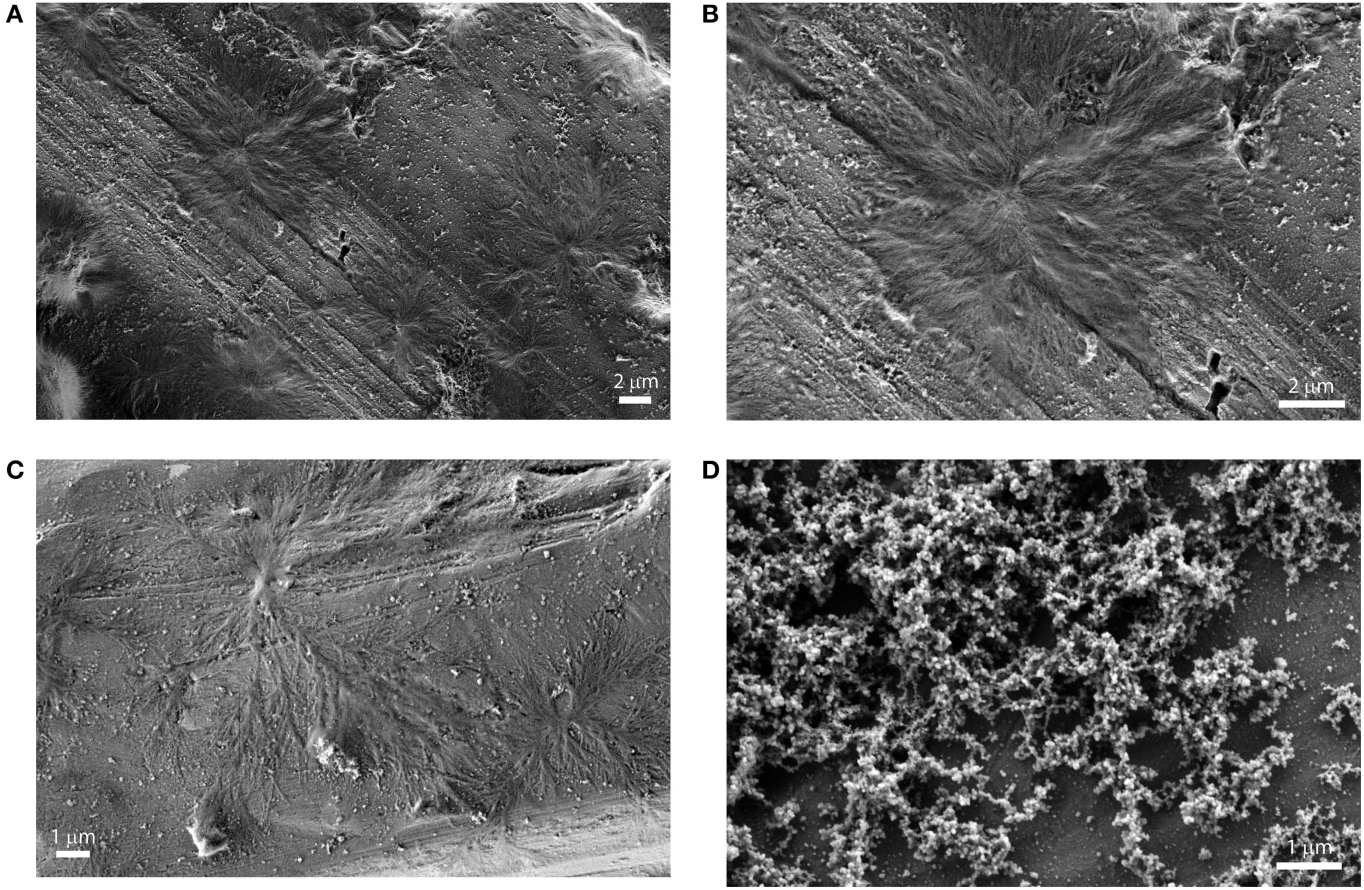
SEM images of drop casted equilibrium solutions of porphyrin aggregates (5 μM (EtOH/H_2_O 25:75 v:v). **(A)**: **(L)ZnP(–)**; **(B)**: **(L)ZnP(–)** enlargement of 5A; **(C)**: **(D)ZnP(–)**; **(D)** racemic mixture.

The structural features of the supramolecular species, have been investigated by Scanning Electron Microscopy (SEM) of drop-casted equilibrium solutions. Micrographies show the formation of regular finely branched fractal structures of about 5–10 μm of diameter, throughout the surface investigated, for both the **(L)ZnP**(**–**) and **(D)ZnP**(**–**) derivatives (Figures 5A–C), in agreement with the peculiar kinetic process found for the formation of these supramolecular self-assembled chiral structures. Interestingly, racemic mixtures resulted in the formation of small globular structure only (Figure 5D), according to the negligible stereospecificity of the supramolecular assemblies. We previously found in fact that, in peptide self-assembly, globular structures predominate when the process is driven by non-specific interactions (e.g., hydrophobic effect). Where specific interactions between ordered structures predominate, the formation of fibrillar structures is then observed (Caruso et al., 2013).

## Conclusions and Future Perspectives

In summary, the self-assembly process of amphiphilic porphyrin derivatives, functionalized by the two different stereoisomers of proline, results in fractal architectures featuring specular chiroptical properties, and alike morphology. Combined spectroscopic and kinetic studies pointed out the occurrence of a two-step process, in which an initial fast and chaotic assembly is followed by a slower stereospecific growth of fractal architectures, featuring intense coupled CD bands. Careful choice of experimental conditions gave a good control on the spectroscopic and morphological features of the final species. Detailed Scanning Electron Microscopy studies confirmed the complex peculiar structures of the obtained species. These findings would be of importance for understanding the fundamental process of evolution of homochirality at biological level (Guijarro and Yus, 2009), and for the development of chiral materials that, coupled with nanotechnology, constitutes a deeply pursued target with a wide range of exploitation in many important areas such as catalysis (Yoon et al., 2012), non-linear optics (Valev et al., 2014), materials science (Wang et al., 2013), sensors (Paolesse et al., 2011; Labuta et al., 2015; Intrieri et al., 2018), and catalysis (Zhou et al., 2018).

## Experimental Section

### General

Reagents and solvents were of commercial sources, in the highest degree of purity and were used as received. (D)-proline *tert*-butyl ester was purchased from Chem Impex International, Inc (USA). Thin-layer chromatography (TLC) was performed on Merck silica gel plates. Chromatographic purification on column was accomplished by using silica gel 60 (70–230 mesh, Sigma Aldrich) as the stationary phase. ^1^H NMR spectra of the new porphyrin derivatives **(D)H**_**2**_**P(-)** and **(D)ZnP**(**–**) were recorded with a Bruker AV300 spectrometer (300 MHz) in CDCl_3_ and THF-d_8_ and are internally referenced to residual proton solvent signals (CDCl_3_: δ = 7.24 ppm, THF-d_8_: δ = 1.73 and 3.58 ppm). FAB mass spectra were obtained with a VG-Quattro spectrometer in the positive-ion mode by using CHCl_3_ as the solvent and m-nitrobenzyl alcohol (Sigma Aldrich) as the matrix. Solvents used for spectroscopic measurements were of spectroscopic grade. UV/Vis spectra were recorded on a Cary 100 spectrophotometer.

CD spectra were performed on a JASCO J-1500, equipped with a thermostated cell holder set at 298 K, and purged with ultra-pure nitrogen gas. Linear Dichroism contribution (LD) has been found to be in all of the cases examined <0.0004 DOD units, in all of the case examined. Fluorescence emission and Resonance Light Scattering measurements were performed on a Fluoromax-4 (HORIBA Scientific) spectrofluorimeter, in a synchronous scan mode, in which the emission and excitation monochromators are pre-set to identical wavelengths. Microstructural analysis of (D)- and (L)- porphyrin aggregates was carried out by using a Field Emission Scanning Electron Microscope FE-SEM, SUPRATM 35, Carl Zeiss SMT, Oberkochen, Germany. Samples have been prepared by slow evaporation of 20 μL of the proper solution, on a previously cleaned aluminum stub (MeOH; N_2_ flow). Sonication of the prepared solutions have been performed by ultrasound thermo-bath Fisher Scientific FB 15047, 90 W power supply, ultrasonic frequency of 37 kHz. Chiral HPLC analyses were performed with a JASCO PU-1580 intelligent HPLC pump equipped with a Rheodyne model 7725i 20 μL injector and coupled with a Varian 2,550 UV detector. Data were collected by using the Borwin software (Jasco, Japan). The Lux i-Amylose-1 [amylose tris(3,5-dimethylphenylcarbamate)] chiral chromatographic column (250 mm × 4.6 mm I.D., 5 μm particle size) was purchased from Phenomenex (Torrance, CA, USA). HPLC runs were performed eluting with a mixture *n*-Hexane:EtOH (80:20, v/v) at flow rates of 0.7 mL min^−1^ and monitoring by UV detection at 350 nm. Samples were dissolved in the eluent, briefly sonicated, and filtered through a 0.45 μm membrane (Whatman, UK) prior to injection.

### Synthesis and Characterization of Porphyrin Derivatives

The chiral porphyrin derivatives investigated were prepared as outlined in Scheme 1. The synthesis and characterization of the (L)-prolinated porphyrins was recently reported (Stefanelli et al., 2019). Thus, herein we report the experimental details to prepare the (D)- counterpart. As expected, the spectroscopic characterization of these new compounds is fully in agreement with those just reported for the corresponding (L)-analogs.

#### Synthesis of (D)H_2_P(–)

To a stirred solution of 5-(4-carboxyphenyl)-10,15,20-triphenylporphyrin (**pCTPP**) (104 mg, 0.158 mmol) in dry CH_2_Cl_2_ (25 mL) kept at 0°C, 1-ethyl-3-(-dimethylaminopropyl)carbodiimide hydrochloride (EDCl) (30 mg, 0.156 mmol), hydroxybenzotriazole (HOBT) (22 mg, 0.162 mmol) and N-methylmorpholine (82 μL, 0.746 mmol) were added. The resulting solution was stirred under N_2_ for 1 h, then (D)-proline *tert*-butyl ester (97μL, 0.474 mmol) was added. The ice bath was removed and the reaction was allowed to reach the room temperature, then stirred for 48 h. Afterwards, the solvent was evaporated and the reaction crude was dissolved in chloroform and washed with brine (3 × 100 mL). The organic phases were collected, dried over anhydrous Na_2_SO_4_ and then purified on silica chromatographic column, eluting with CHCl_3._ In a second step, the obtained porphyrin was dissolved in TFA/CH_2_Cl_2_ mixture (2/3 v/v; 25 mL). The reaction was stirred at room temperature for 1.5 h. After the solvent removal under reduced pressure, the green residue was dissolved in CHCl_3_ and neutralized with a saturated solution of NaHCO_3_. The organic phase was dried over Na_2_SO_4_, concentrated and crystallized by adding an equal volume of n-pentane. Porphyrin was obtained as bright purple crystals (81 mg, 0.107 mmol; 68% yield). UV-Vis (CHCl_3_): λ_max_ (ε, M^−1^ cm^−1^) = 419 (125,900), 515 (6,310), 547 (6,290), 585 (5,010), 640 (3,160) nm. ^1^H NMR (300 MHz, CDCl_3_): 8.80 (br s, 8 H, β-pyrr), 8.19 (m, 4H, ArH), 8.17 (m, 6H, ArH), 7.71 (m, 3H, ArH), 7.60 (m, 6H, ArH), 4.5 (m, 1 H, proline α-H), 3.97 (m, 2 H, proline δ-H), 2.44 (m, 2 H, proline β-H), 2.07 (m, 2 H, proline γ-H), −2.79 (s, 2H, NH). FAB-MS (NBA), m/z: 756 [M]^+^.

#### Synthesis of (D)ZnP(–)

To a stirred solution of porphyrin **(D)H**_**2**_**P(–)** (90 mg, 0.119 mmol) dissolved in CHCl_3_ (15 mL) an excess of a methanolic solution of Zn(OAc)_2_·2H_2_O (43 mg, 0.234 mmol dissolved in 3 mL of MeOH) was added. The progress of metal insertion was followed by UV-Vis spectroscopy (Soret and Q visible band changes). After 1 h of stirring at room temperature, the complex was quantitatively formed and obtained as a cherry-purple solid after solvent evaporation. Pure purple crystals of the desired compound was obtained after crystallization from CHCl_3_/n-pentane (93 mg, 0.114 mmol; 96% yield). UV-Vis (THF): λ_max_ (ε, M^−1^ cm^−1^) = 424 (451,000), 556 (15,500), 595 (5,600) nm. ^1^H NMR (300 MHz, THF-*d*_8_): 8.83 (m, 8 H, β-pyrr), 8.21 (m, 4H, ArH), 8.19 (m, 6H, ArH), 7.76 (m, 3H, ArH), 7.65 (m, 6H, ArH), 3.99 (m, 1 H, proline α-H), 3.89 (m, 2 H, proline δ-H), 2.34 (m, 2 H, proline β-H), 2.18 (m, 2 H, proline γ-H). FAB-MS (NBA), m/z: 818 [M]^+^

#### Determination of Enantiomeric Purity of (D)- and (L)ZnP(–)

The enantiomeric purity of **(D)ZnP(**–**)** and **(L)ZnP(**–**)** was checked by HPLC chromatography on a chiral stationary phase (c.s.p.), a method which has proved to be efficient for enantioseparation of chiral tetrapyrrole macrocycles (Belviso et al., 2018a,b). Accordingly, the racemic mixture and the two enantiomers were eluted on amylose tris(3,5-dimethylphenylcarbamate) c.s.p. This c.s.p. allowed separation of the racemic mixture into the two enantiomers **(D)ZnP**(**–**) and **(L)ZnP**(**–**) (see Supplementary Figure 4) even if a peak overlap occurs as a consequence of a peak tailing probably due to aggregation phenomena in the required elution mixture. In particular, **(L)ZnP**(**–**) showed a retention time of 28.90 min, while **(D)ZnP**(**–**) a retention time of 36.70 min. Comparison of the racemic mixture chromatogram with those of the single enantiomers allowed to establish that both samples were enantiomerically pure, showing no traces of the other stereoisomer.

### Aggregation Studies

All the spectroscopic studies have been carried out at 298 K. Solutions suitable for the aggregation studies were prepared as follows. Porphyrin stock solutions in ethanol (*ca*. 10^−4^ M concentration) were gently warmed, then briefly sonicated and finally filtered through a 0.22 μm Nylon® membrane (HahnemÃ1/4le Albet® Syringe Filters), prior to use. These precautions have been taken to avoid uncontrolled nucleation of porphyrin protoaggregates that would affect the reproducibility of the experiments. Check of the effective concentration was made by UV-Vis spectroscopy in pure ethanol (Soret band intensity of the porphyrins in monomeric form; ε = 4.51 × 10^5^ M^−1^ cm^−1^). Aggregation of title porphyrins has been carried out in aqueous ethanol solutions (EtOH/H_2_O 25:75 v:v; 298 K) at 1, 5 and 10 μM concentration, by strictly following a “porphyrin first” protocol. Namely, a proper aliquot of a stock solution of porphyrin (10–100 μL), was added to the required amount of ethanol (final volume of 1.0 mL) in an 8 mL glass vial, and briefly sonicated. To this solution 3.0 mL of water were then slowly added, again under sonication, to give 4.0 mL of resulting solution with 25% v:v solvent proportion, with the required porphyrin concentration. A ca. 2.5 mL portion was finally transferred into a quartz cuvette and the relative spectra were then acquired at different time in order to monitoring the temporal evolution of the systems. This second sonication step appears to be of crucial importance for a good reproducibility of the results, that depends on the homogeneity of the morphology of the initially formed porphyrin clusters, which would be detrimentally affected by the unavoidable occurrence of both gradient of concentration and solvent polarity changes, during the mixing with water. The porphyrin stock solutions should be used within 2 weeks from preparation, to ensure optimal reproducibility of the results.

### Kinetic Studies

Kinetic studies on the aggregation process of the title porphyrins, have been carried out in EtOH/H_2_O (75:25 v:v) and followed by CD spectroscopy. In all of the case examined, the first aggregation stage (Type-I aggregate formation) was in general too fast to be followed by conventional spectroscopic techniques. As far as the second process is concerned (Type-II aggregate formation) autocatalytic-type behavior of the molar ellipticity [θ] *vs*. time profiles have been obtained. The experimental data have been fitted by the Equation 1, reported in the Results and Discussion section of the text. The kinetic parameters were obtained by non-linear regression fit (KaleidaGraph 4.1, Synergy Software, 2011), over tens of data points. Entry values of *k* and *n* were initially obtained by running the program with fixed initial and equilibrium experimental values (theta molar or extinction). Final regressions were operated with “free” initial and final parameters to give final calculated values, that have been found to be always in good agreement with the experimental ones. The final kinetic data, reported in Table 1, are the averaged values obtained over at least three different runs, and for both minimum and maximum wavelength of lower energy, most intense coupled bands at 436 and 443 nm, of the CD spectra.

## Data Availability Statement

The original contributions presented in the study are included in the article/Supplementary Material, further inquiries can be directed to the corresponding author/s.

## Author Contributions

DM contributed to conception and design of the study. MSt synthesized the title porphyrins. FZ performed SEM characterization of samples. DM, GMag, and MSa performed the Circular Dichroism measurements. MSa and MV performed the fluorescence spectroscopy studies. SB, GMar, and SS performed chiral HPLC analysis. DM organized the database and performed the fitting and statistical analysis. GMag performed the editing of the figures and plots. MSt, DM, MV, CD, SS, and RP wrote sections of the manuscript. RP was responsible for the financial support to the work. All authors contributed to the discussion and interpretation of the results and to manuscript revision, and read and approved the submitted version.

## Conflict of Interest

The authors declare that the research was conducted in the absence of any commercial or financial relationships that could be construed as a potential conflict of interest.
