# Supplementary material for: Porphyrins Through the Looking Glass: Spectroscopic and Mechanistic Insights in Supramolecular Chirogenesis of New Self-Assembled Porphyrin Derivatives
Source: Front Chem. 2020 Oct 15;8:587842. doi: 10.3389/fchem.2020.587842 (PMC7593786; doi:10.3389/fchem.2020.587842)
Supplement: Supplementary file 1 [file Data_Sheet_1.PDF]

Supplementary Material

1 Effect of sonication on the solvent driven aggregation (*a bit of history …*).

In the early stage of this work we consistently found, in a reproducible way, a different evolution of the aggregation process of the **(L)ZnP(-)** derivative, with respect to its **(D)ZnP(-)** enantiomer. Both of the monomers presented similar biphasic aggregation behavior, but the final structures of the (L)-proline decorated platform featured more intense CD spectral pattern, by about one order of magnitude, even with substrates of different synthetic batches.

An initial explanation for these unexpected results was tentatively interpreted by us on the basis of the peculiar structure of the self-assembled fractal-type species. These fascinating randomly growing architectures, are *incidentally chiral* in nature also if constituted of achiral building blocks (Nieckarz, Szabelski, 2016; Shang et al, 2015; Katzenelson et al, 1996), and their enantiomers could not be obtained in practice (*virtual* enantiomorphism), with their *degree of chirality* scaling with their size (i.e. size-dependent *functional chirality*; Katzenelson, Avnir, 2000). Based on these properties and on our experimental results we deduced that i) the chiral information brought by the proline residues could be read out in *reversed chirality mode* only in the case of the initial formation of small-sized, "*quasi-enantiomeric* structures" (i.e. Type-I aggregates), which consequently feature specular CD signals and alike *g*-factors; ii) in the subsequent stage the formation of larger structures (Type-II aggregates) would cause, after a certain threshold, a divergence from specularity, resulting in an unbalancing in the crucial reading-out step of the chiral information brought by the incoming L- or D-prolinate moieties.

On the light of the "*beneficial*" effect of the crucial sonication step introduced in the preparation of the solutions, that notably brought the aggregative process to the expected identical behavior, another hypothesis on the occurrence of the precedent observed differences can be now offered. The actual interpretation would consider the occurrence of polymorphic species in solid state, which may have different kinetic of solubilization. The different solubility would lead to the presence of small, even below the level of spectroscopic detection, proto-aggregation nuclei which would differently drive the kinetic and spectroscopic outcome of the process. These differences, although minimal and within the experimental errors at early stage (Type-I species), would bring to large differentiations in the chiral amplification after the subsequent aggregate growth (Type-II structures). This should be a consequence of the nature of the chiral functionality bound to the periphery of the macrocycles. It is well known, in fact, that the proline ring undergoes in solution an *E* to *Z* isomerization equilibrium, with the *E* form usually more stable for steric reasons. It has been found, however, that in the case of surfactants bearing an anionic prolinate group as a charged head, the conformational equilibrium lead to the less favored isomer (*Z*) under aggregative conditions, i.e. at surfactant concentration above the *cmc* (Borocci et al, 1999), as a consequence of headgroups hydration, molecular recognition, and also water penetration.

In our case, as also witnessed by the inversion of the signs of CD bands on the aggregates (see Main Text), an additional effect of the coordination to the core Zn(II) ion would be taken into consideration. This would bring to different domains of the enantiomeric macrocycles in the solid state causing, finally, different kinetic of solubility.

The effect of the sonication should be to re-establish the homogeneity of the initially formed enantiomorphic species.

Crystallographic studies aimed at the elucidation of this important issue, although not central for our present study, are actively under way and the results will be published elsewhere.

2 Supplementary Figures

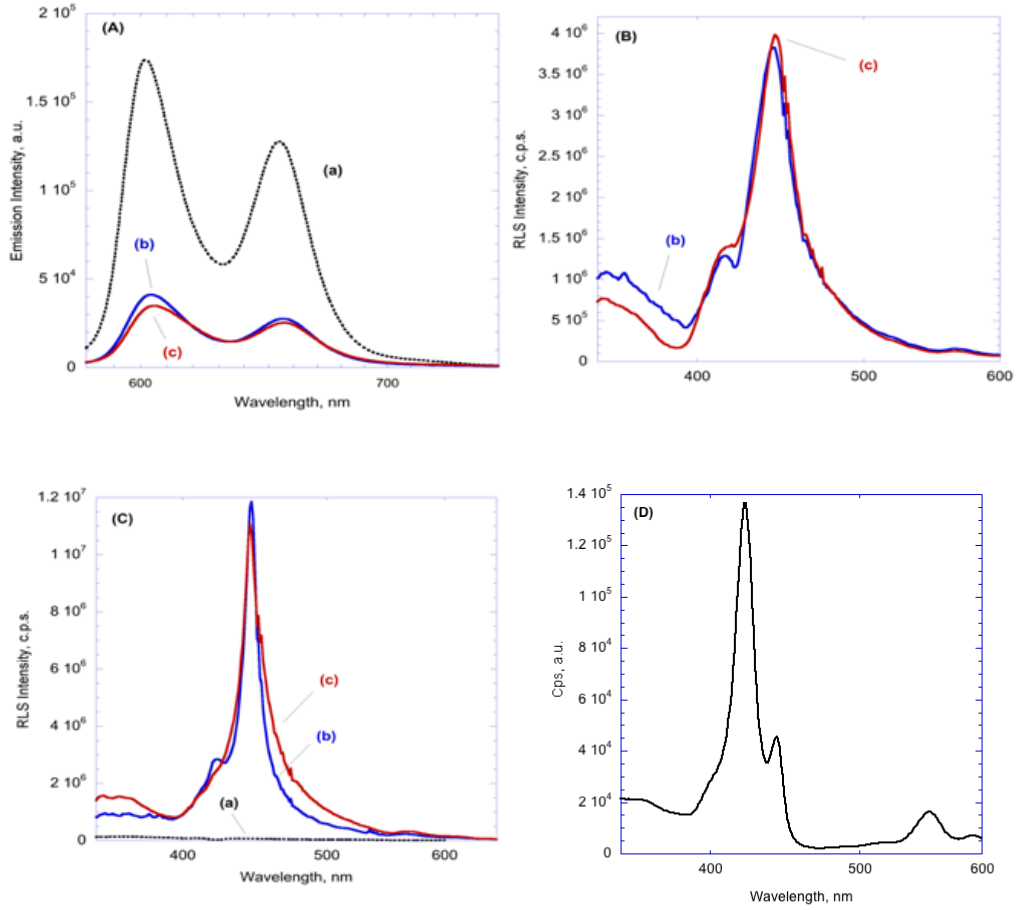

Supplementary Figure S1. (A) Fluorescence emission spectra of **(L)H_2P(-)** 5.0 µM, 298 K, in a) monomeric form (EtOH); λ_{exc} = 560 nm, and as Type-II aggregates (EtOH/H_2O 25/75 v:v), λ_{exc} = 443 nm: b) **(L)H_2P(-)** 5.0 µM; c) **(D)H_2P(-)** 5.0 µM. (B) RLS spectra of Type-I aggregates (5.0 µM; EtOH/H_2O 25/75 v:v, 298 K): b) **(L)H_2P(-)** (blue trace) and c) **(D)H_2P(-)** (red trace). (C) RLS spectra of Type-II aggregates (5.0 µM; EtOH/H_2O 25/75 v:v, 298 K): a) **(L)H_2P(-)** (monomeric form, black dotted trace) b) **(L)H_2P(-)** (blue trace) and c) **(D)H_2P(-)** (red trace). (D) Excitation spectra (λ = 650 nm) of **(L)H_2P(-)** Type-II aggregates (5.0 µM; EtOH/H_2O 25/75 v:v, 298 K).

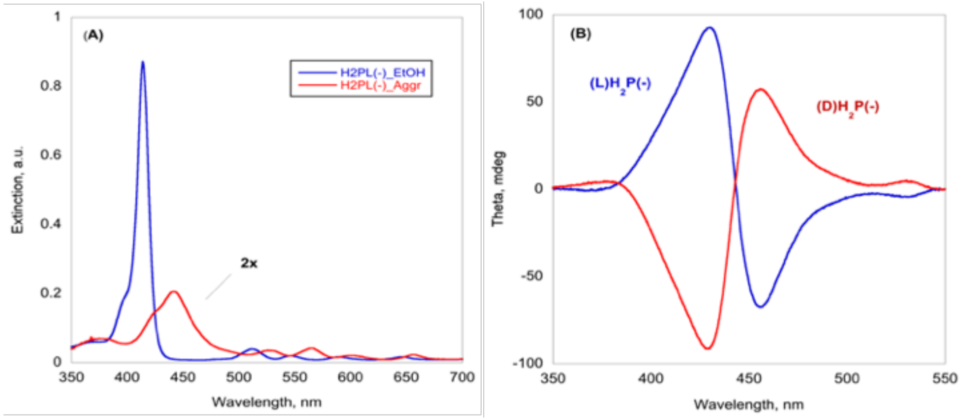

Supplementary Figure S2. (A) UV-Vis spectral changes of **(L)H$_2$P(-)** 5.0 µM in monomeric form (EtOH; blue line) and as aggregates (EtOH/H$_2$O 25/75 v:v; red line). (B) CD spectra of equilibrium solution of aggregates of **(D)H$_2$P(-)** (red trace) and **(L)H$_2$P(-)** (blue trace); 5.0 µM, EtOH/H$_2$O 25/75 v:v; 298 K.

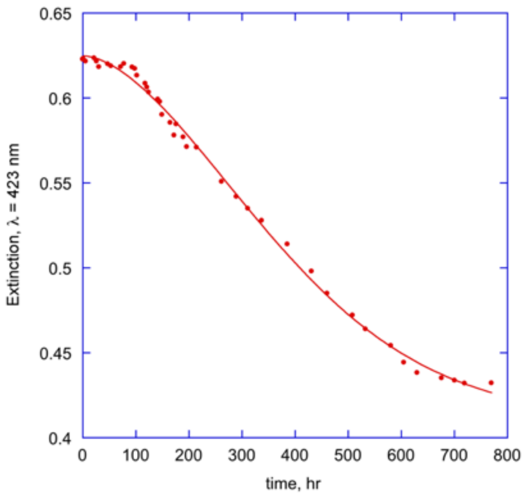

Supplementary Figure S3. UV-Vis aggregation kinetic (Type-II aggregates) of **(L)H$_2$P(-)** 5.0 µM, EtOH/H$_2$O 25/75 v:v; 298 K, at λ = 423 nm. The solid line is the theoretical fit according to Eq. 1.

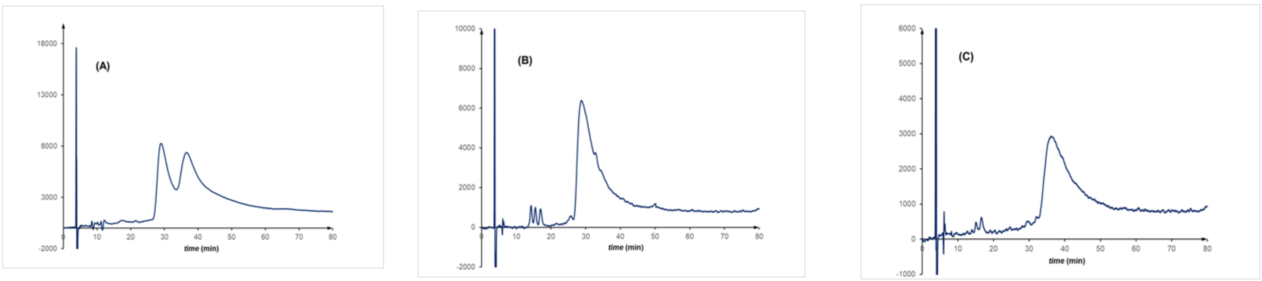

Supplementary Figure S4. HPLC separation of **(D)- and (L)ZnP(-)**. Column: Lux i-Amylose-1 250

mm × 4.6 mm (5 μ); mobile phase: *n*-hexane:EtOH (80:20, v/v); flow rate: 0.7 mL min–1. UV detection at 350 nm. T = 25°C. (A) Racemic mixture. First enantiomer Tr1 = 28.90 min, second enantiomer Tr2 = 36.70 min. (B) **(L)ZnP(-)**, Tr = 28.95 min. (C) **(D)ZnP(-)**, Tr = 36.80 min.

3 References

Borocci, S., Mancini, G., Cerichelli, G. (1999). Conformational behavior of aqueous micelles of sodium N-dodecanoyl-L-prolinate. Langmuir, 15, 2627-2630. DOI: 10.1021/la980988z

Nieckarz, D, Szabelski, P. (2016). Chiral and fractal: from simple design rules to complex supramolecular constructs. Chem. Commun. 52, 11642-11645. DOI: org/10.1039/C6CC05348C

Katzenelson, O., Zabrodsky Hel-Or, H., Avnir, D. (1996). Chirality of large random supramolecular structures. Chem. Eur. J. 2, 174-181.

Katzenelson, O., Avnir, D. (2000). Quantitative chirality/enantioselectivity relations in large random supramolecular structures. Chem. Eur. J. 6, 1346-1354.

Shang. J., Wang, Y., Chen, M., Dai, J., Zhou, X., Kuttner, J., Hilt, G. et al, (2015). Assembling molecular Sierpinski triangle fractals. Nat. Chem. 7, 389-393. DOI: 10.1038/nchem.2211
