# Supplementary figures and images for: Porphyrins Through the Looking Glass: Spectroscopic and Mechanistic Insights in Supramolecular Chirogenesis of New Self-Assembled Porphyrin Derivatives
Source: Front Chem. 2020 Oct 15;8:587842. doi: 10.3389/fchem.2020.587842 (PMC7593786; doi:10.3389/fchem.2020.587842)

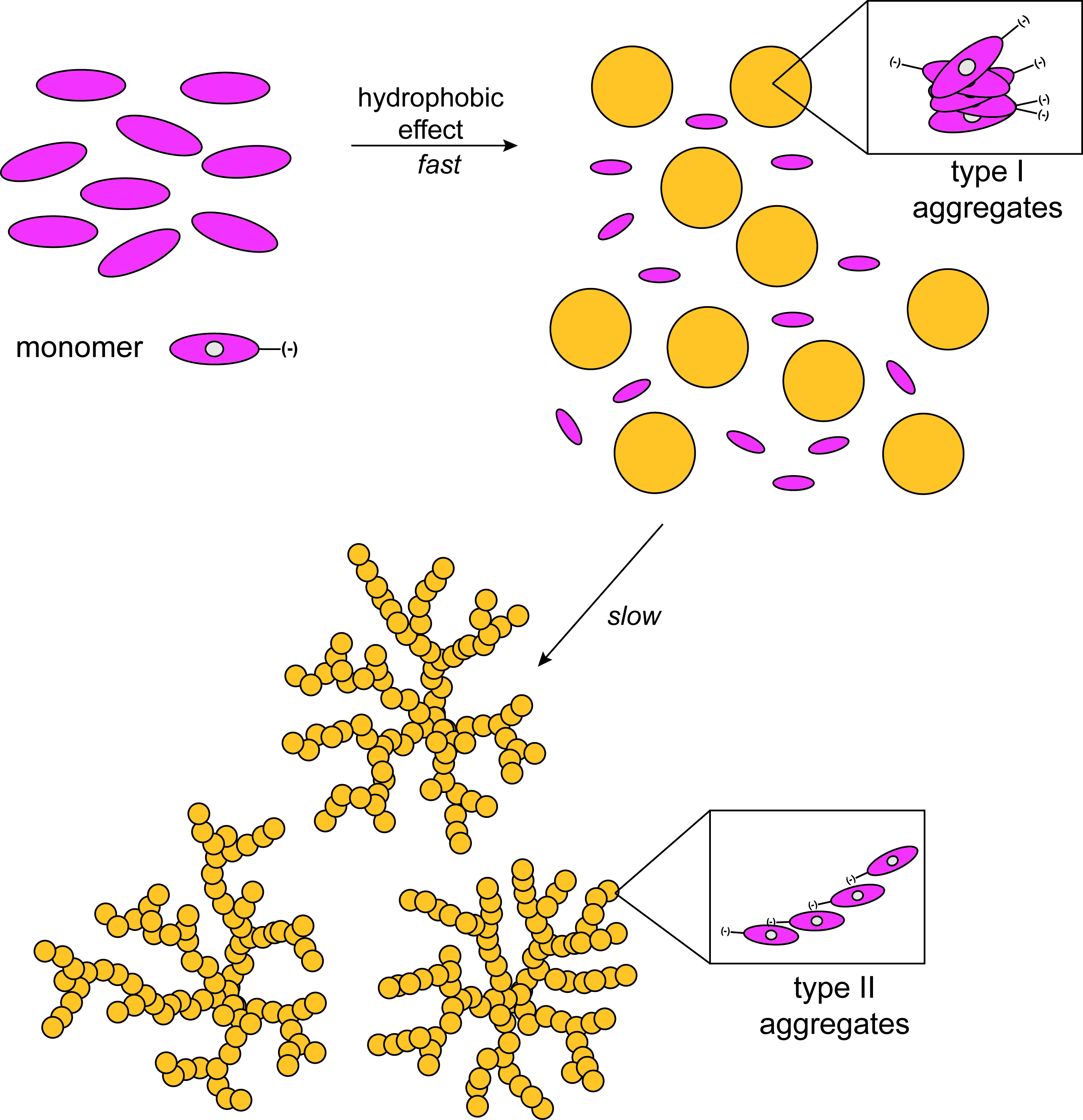

Supplement: Supplementary file 2 [file Image_1.TIF]
